# Comprehensive Characterization via Molecular Imaging, Longitudinal Multisite Sampling, and Autoptic Work-up in Advanced Small Cell Lung Cancer Undergoing SSTR-Directed Radiopharmaceutical Therapy

**DOI:** 10.2967/jnumed.124.268513

**Published:** 2025-02

**Authors:** Johanna S. Enke, Nic G. Reitsam, Sebastian Dintner, Friederike Liesche-Starnecker, Tina Schaller, Josua A. Decker, Angela Langer, Eva Sipos, Ana Antic Nikolic, Thomas Kröncke, Martin Trepel, Constantin Lapa, Rainer Claus, Bruno Märkl, Ralph A. Bundschuh

**Affiliations:** 1 Nuclear Medicine, Faculty of Medicine, University of Augsburg, Augsburg, Germany;; 2 Pathology, Faculty of Medicine, University of Augsburg, Augsburg, Germany;; 3 Bavarian Cancer Research Center (BZKF), Augsburg, Germany;; 4 Comprehensive Cancer Center, Faculty of Medicine, University of Augsburg, Augsburg, Germany;; 5 Radiology, Faculty of Medicine, University of Augsburg, Augsburg, Germany;; 6 Augsburg Central Biobank, Faculty of Medicine, University of Augsburg, Augsburg, Germany; and; 7 Hematology and Oncology, Faculty of Medicine, University of Augsburg, Augsburg, Germany

**Keywords:** small cell lung cancer, radiopharmaceutical therapy, somatostatin receptor, autopsy, tumor heterogeneity

## Abstract

Despite the addition of immune checkpoint blockade to first-line chemotherapy, the prognosis for patients with small cell lung cancer (SCLC) is still devastating. For the subset of SCLC with somatostatin receptor (SSTR) overexpression, radiopharmaceutical therapy (RPT) might be an effective future treatment option. **Methods:** Here, we present the case of a heavily pretreated stage IV SCLC patient showing an exceptional response to SSTR-directed RPT. A comprehensive translational work-up consisting of histopathologic, immunohistochemical, and molecular pathology analyses at different time points during treatment and especially of lesions with discordant tracer uptake was performed. **Results:** Besides a promising response to RPT, interesting signs of clonal dynamics under therapy and, most importantly, SSTR downregulation of some lesions as a potential evasion mechanism to SSTR-directed RPT could be identified. **Conclusion:** This unique investigation for a clinical–molecular understanding of novel treatment paradigms in SCLC may provide the basis for future treatment designs.

## Visual Abstract

**Figure:**
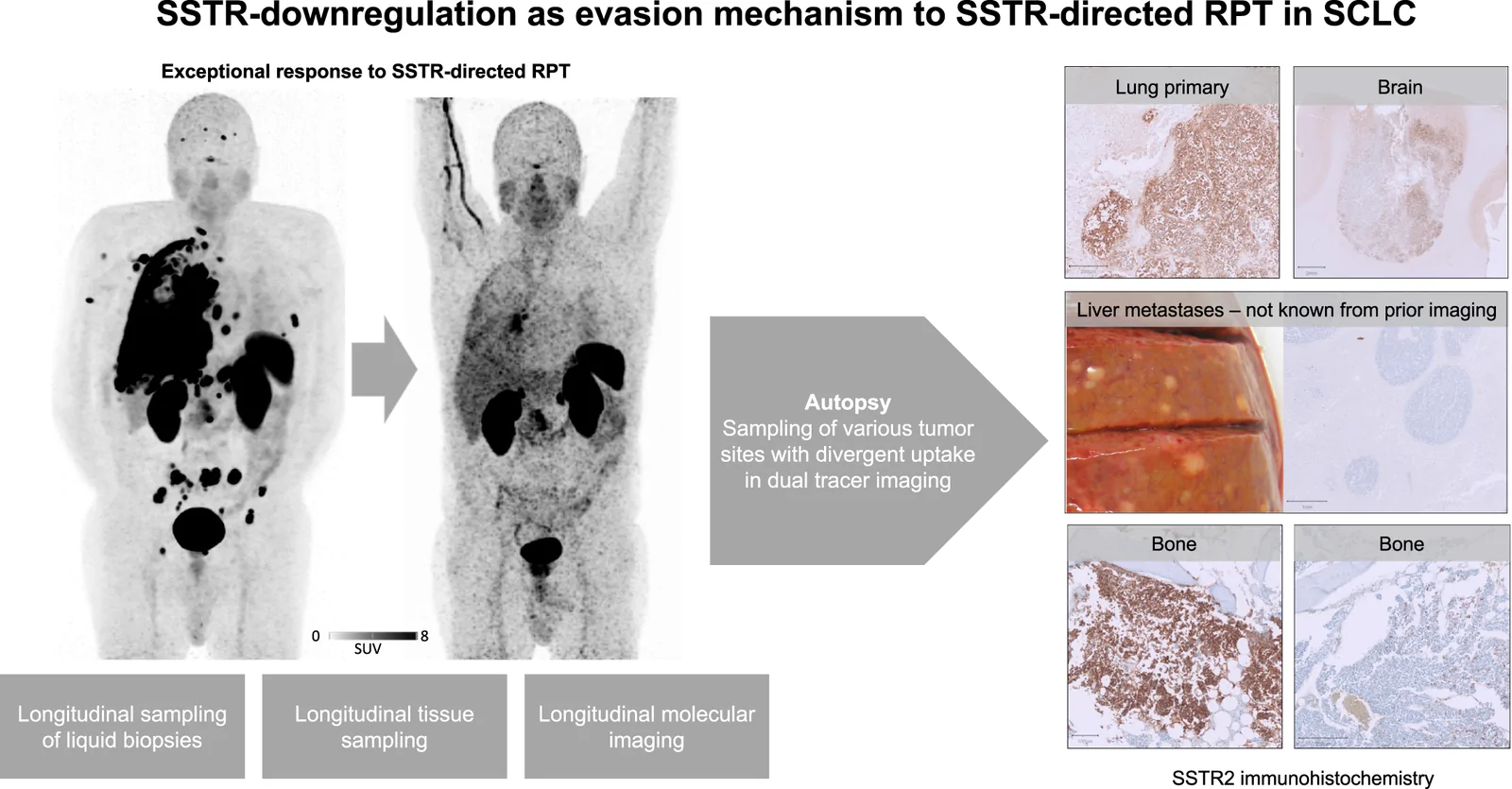

Small cell lung cancer (SCLC) is an aggressive malignancy associated with a poor prognosis and early treatment resistance (1). Despite multimodal therapy regimens including chemotherapy, external radiation therapy, and immunotherapy (2), the 5-y overall survival rate remains extremely poor. Recently, our understanding of SCLC as a biologically heterogeneous disease has grown, leading to the delineation of distinct molecular subtypes (3). Somatostatin receptor (SSTR) overexpression has been reported in a substantial subset of SCLC (4) as a rationale for a SSTR-directed radiopharmaceutical therapy (RPT), with, so far, inconclusive results. SSTR-directed RPT is a targeted treatment option for SSTR-overexpressing tumors. Most experience has been gained from neuroendocrine neoplasms of the gastroenteropancreatic system. Recently, the NETTER-1 and NETTER-2 trials were able to demonstrate significant benefits in terms of progression-free survival, objective response rate, and health-related quality of life after the administration of 4 cycles of 7.4 GBq of SSTR-directed RPT (5–7).

Here, we present the paradigmatic case of a heavily pretreated 64-y-old man with stage IV SCLC showing an exceptional treatment response to SSTR-directed PRT. The patient agreed before his death to an autopsy and consecutive work-up, enabling us to investigate the SCLC evolution and treatment effects of SSTR-directed RPT at different time points and in different tumor manifestations with diverging tracer uptake. Hence, we conducted translational investigations of longitudinal tissue and serum sampling (Augsburg Longitudinal Plasma Study for the Evaluation of Liquid Biopsy as Diagnostic Tool (8), NCT0524513), autopsy material, and multiple-time-point dual-tracer PET/CT imaging.

With clinical trials currently assessing the potential of SSTR-directed RPT in SCLC (NCT05142696), we firmly believe that our analyses provide an important step toward a better understanding of the tumor biology and escape mechanisms in SCLC undergoing RPT.

## MATERIALS AND METHODS

### Imaging and RPT

Before and during RPT, the patient underwent PET/CT imaging; a detailed description can be found in Supplemental Table 1 (supplemental materials are available at http://jnm.snmjournals.org). Image acquisition and interpretation were performed according to current guidelines (9).

The patient received 7 cycles of SSTR-directed RPT with a mean intravenous activity of 7.34 GBq per cycle (range, 4.5–9.8 GBq) approximately every 4 wk (Supplemental Table 2). Each cycle was administered after prior treatment with antiemetic and nephroprotective medication (9). Adverse events were assessed according to Common Terminology Criteria for Adverse Events, version 5.0 (9).

### Ethics Approval and Patient Consent

Before his death, the patient consented to autopsy and further work-up of his case.

The patient was part of the ALPS trial, which is conducted in accordance with the Declaration of Helsinki and approved by the local ethics committee (Augsburg Longitudinal Plasma Study for the Evaluation of Liquid Biopsy as Diagnostic Tool, NCT0524513) (8).

### Histopathology, Immunohistochemistry, and Molecular Pathology

Further details on histopathology, immunohistochemistry, and molecular analysis of tissue and circulating tumor DNA can be found in the supplemental materials (10–14).

## RESULTS

### Clinical Course

**FIGURE 1.**
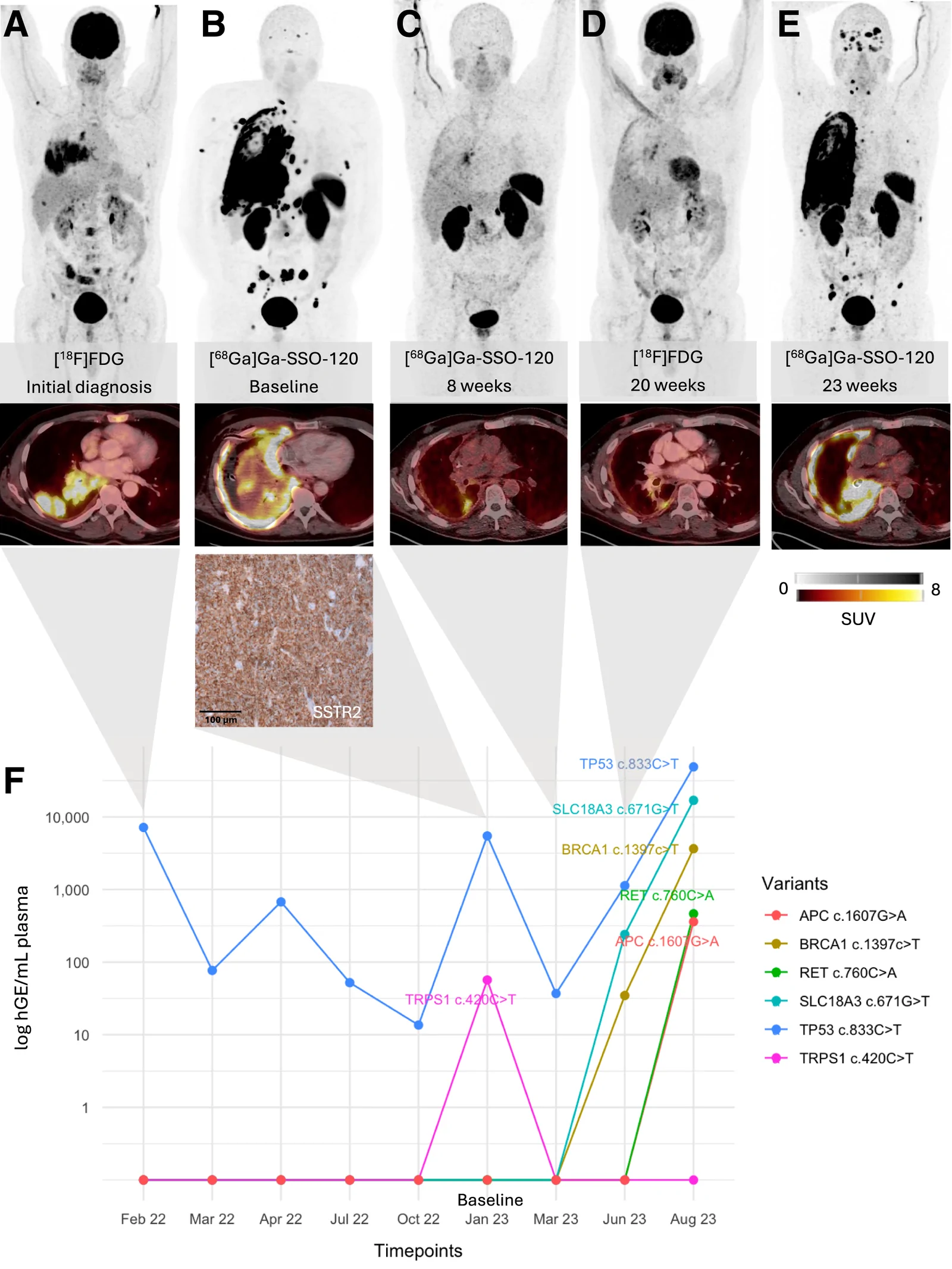
Longitudinal molecular imaging and profiling of circulating tumor DNA in stage IV SCLC patient reveals sensitive monitoring of disease burden and dynamic clonal evolution. (A–E) Maximum-intensity projections and axial images of primary tumor in right lung with different tracers over course of disease, with initial response to therapy and immunohistochemistry of SSTR2 at baseline imaging. (F) Circulating tumor DNA quantification, longitudinal changes of mutated alleles (logarithmic scale of haploid genome equivalents [hGE] per milliliter of plasma), and clonal composition over disease course from start of initial therapy in February 2022 to time of death in August 2023.

Before referral to the nuclear medicine department, the patient received 3 therapy lines over the course of 1 y (first line: atezolizumab, carboplatin, and etoposide; second line: topotecan; third line: epirubicin, cyclophosphamide, and vincristine) but experienced progressive disease. A schematic timeline of events is displayed in Supplemental Figure 1. Because of extensive SSTR positivity (as determined by PET) of all tumor manifestations, namely the extensive primary tumor with pleural metastases, multiple involved mediastinal lymph nodes and in the upper abdomen and disseminated bone and brain metastases (Fig. 1B; Supplemental Figs. 2B1–2B5), the patient was deemed eligible for SSTR-directed RPT (15). After 2 therapy cycles, the patient presented with a partial response, with a significant reduction of the primary tumor, pleural metastases, and lymph node metastases and only minimal residual metabolic activity in the bone metastases (Fig. 1C; Supplemental Figs. 2C1–2C5). No relevant toxicity according to Common Terminology Criteria for Adverse Events, version 5.0, could be noted. Furthermore, the patient’s quality of life significantly improved in terms of physical ability and reduced side effects compared with prior chemotherapy regimens. After administration of cycle 4, [^18^F]FDG PET/CT still displayed an overall stable total tumor burden (Fig. 1D) except for 2 new bone lesions in the lower spine. SSTR-directed PET/CT 3 wk later (application of cycle 5 was delayed for personal reasons) revealed a significant progression of the disease, with revitalized and new tumor manifestations (primary tumor, brain, bone, and lymph nodes) (Fig. 1E; Supplemental Figs. 2E1–2E5). However, as all tumor manifestations remained SSTR-positive, another 3 cycles of RPT could be administered. All further cycles of RPT were coadministered with corticosteroids to prevent increasing cerebral edema of recently progressive brain metastases (symptomatic by generalized seizures; Supplemental Table 2).

**FIGURE 2.**
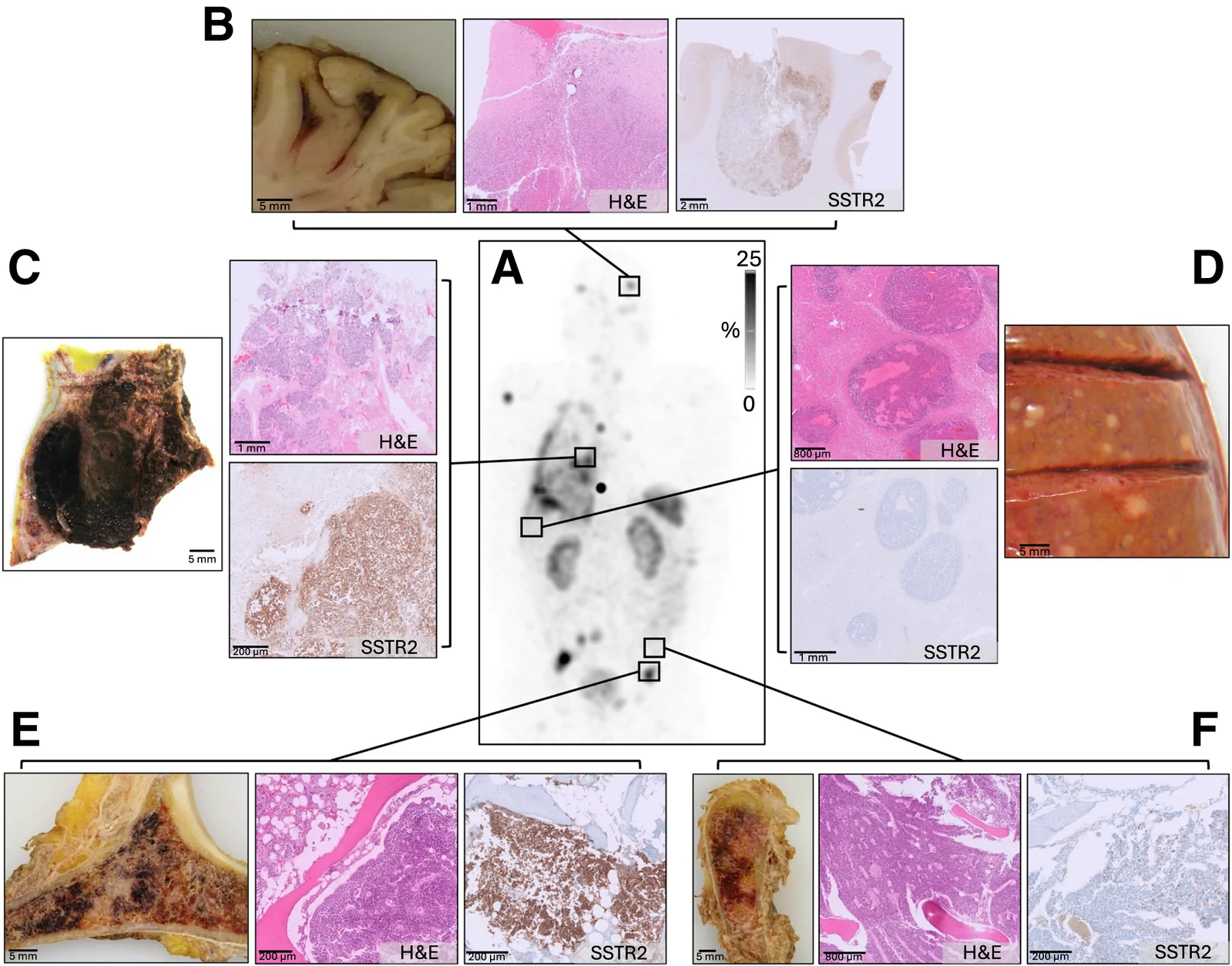
Autopsy findings of different tumor manifestations with divergent SSTR expression on molecular level and in posttherapeutic imaging after cycle 7 RPT. (A) Whole-body posttherapeutic scintigraphy after cycle 7 RPT with accumulation of radiopharmaceutical in known tumor manifestations. (B) Brain metastasis with partly necrotic changes and moderate to strong partial SSTR2 expression. (C) Tumor lesion in right lung with strong SSTR2 expression and some fibrotic areas. (D) Multiple subcapsular liver metastases with loss of SSTR2 expression and partly with central necrosis. (E) Diffuse infiltration of bone marrow with strong SSTR2 expression in acetabulum. (F) Diffuse infiltration of bone marrow of os ilium with loss of SSTR2 expression. H&E = hematoxylin and eosin.

Posttherapeutic scintigraphy of cycle 7 RPT demonstrated stable disease (Fig. 2A) in most tumor manifestations (primary tumor, mediastinal lymph nodes, and brain manifestations), with reduced uptake in some bone lesions, such as the left acetabulum (Supplemental Figs. 2F1–2F5). Three weeks after administration of the last treatment cycle, the patient experienced a rapid worsening of his health and sadly died 24 h later in his home.

### Extensive Morphomolecular Workup

#### Autopsy Reveals Previously Unknown Hepatic and Adrenal Metastases

Central nervous system dysregulation due to cerebral edema with signs of cerebellar tonsillar herniation was the cause of death (brain weight, 1,500 g). Besides several cerebral metastases, extensive neoplastic meningitis as well as carcinoma infiltrates in the trigeminal nerve could be found, which correlates well with the emergence of generalized seizures close to the patient’s death. Additional to previously known multiple tumor manifestations in the brain and bones, autopsy revealed extensive tumor residuals around the primary tumor of the right lung, with infiltration of the chest wall and pleura. Furthermore, autopsy revealed multiple—previously unknown—hepatic lesions and an adrenal metastasis on the left. For further diagnostic work-up, we sampled tumor manifestations with diverging tracer uptake in posttherapeutic imaging. To better identify and localize the bone lesions with divergent tracer uptake (Supplemental Figs. 2F4 and 2F5), postmortem imaging (CT and radiography) was performed (Supplemental Fig. 3).

On histopathologic review, some lesions showed necrotic changes (cerebral), concordant with prior imaging of the brain (Supplemental Table 1), or fibrotic changes (lung), in line with prior reports describing a higher stroma fraction after RPT in pancreatic neuroendocrine tumors (16).

#### Immunohistochemical Analysis Reveals Downregulation of SSTR2 Expression as Potential Evasion Mechanism

We used immunohistochemical surrogate markers (NEUROD1, POU2F3, ASCL1, and YAP1) for SCLC subtyping (13,14). Immunohistochemistry of the primary bronchial tumor lesion and of pleural recurrence enabled us to assign the SCLC to the NEUROD1-dominant subtype (13) (strong NEUROD1 expression, partly moderate ASCL1 expression, YAP1- and POU2F3-negative; Supplemental Fig. 4), which had already been shown to correlate on a molecular level with high SSTR2 expression (3).

The primary lesion (bronchial biopsy), recurrences (pleural biopsy and autopsy tissue), and most metastases (autopsy tissue) showed a strong membranous SSTR2 expression (score, 3+). All liver lesions (≤0.8 cm) that were not known before autopsy and were not highlighted by imaging modalities lacked SSTR2 and NEUROD1 expression but could not be assigned to another molecular subtype via immunohistochemistry (Supplemental Fig. 5). A vital metastasis in the left iliac bone did not show any SSTR2 expression, correlating with the most recent prior posttherapeutic scintigraphy with reduced tracer uptake, whereas intense SSTR expression had been present on prior SSTR-directed PET scans. SSTR2 expression in a small adrenal gland metastasis (∼100 µm) was also reduced (score, 1+). All lesions with missing or weak SSTR2 expression showed vital tumor cells and no specific radiation-induced morphologic changes (no necrosis, no bizarre nuclei) on histopathologic evaluation. None of the lesions showed SSTR5 expression (Supplemental Fig. 6), which is in line with previous findings (17). No relevant upregulation of programmed-death ligand 1 on RPT could be observed (Supplemental Fig. 7).

Immunohistochemical stains are displayed in Figure 2 and Supplemental Figures 4–7.

#### NGS-Based Panel Sequencing Shows Stable Tumor Genetics Under Therapeutic Pressure

NGS-based panel sequencing (TrueSight Oncology 500) was performed on the bronchial biopsy, the pleural recurrence, and several tumor manifestations secured during the autopsy (lung, lymph node, liver, and 2 samples of brain tissue; DNA quality of ethylenediaminetetraacetic acid–decalcified bone material did not allow genetic testing). All analyzed lesions were characterized by a pathogenic *TP53* mutation (*TP53*; exon 8; c.833C>T; p.P278L) with variant allele frequencies ranging from 73% to 95%. These results suggest that the different SCLC tumor manifestations remained comparably stable with regard to their genetics in this case despite therapeutic pressure. The results of the genetic testing are summarized in Table 1.

**TABLE 1.** NGS-Based Molecular Analyses of SCLC Lesions

Sampling material	Lesion	DNA alteration	TMB	MSI	CNV
Diagnostic biopsies	Initial diagnosis (bronchial biopsy)	TP53 (ENST00000269305.4); exon 8; c.833C>T; p.P278L; VAF 94; 675/721; pathogen; cell cycle	0.8 mut/Mb (1.27 Mb)–TMB-low	2.52% (3/119)–MSS	MYCL 1.579 (fold change); 4 (copy number) NRAS 1.823 (fold change); 4 (copy number) MDM4 1.731 (fold change); 4 (copy number) PIK3CB 1.446 (fold change); 4 (copy number) PIK3CA 1.397 (fold change); 4 (copy number) RICTOR 1.622 (fold change); 4 (copy number) FGF10 1.536 (fold change); 4 (copy number) FGF1 1.436 (fold change); 4 (copy number) ERBB2 1.471 (fold change); 4 (copy number) BRCA1 1.501 (fold change); 4 (copy number) RPS6KB1 1.535 (fold change); 4 (copy number)
	Pleural recurrence	TP53 (ENST00000269305.4); exon 8; c.833C>T; p.P278L; VAF 95; 665/699; pathogen; cell cycle	0.8 mut/Mb (1.27 Mb)–TMB-low	4.3% (5/116)–MSS	MYCL 1.407 (fold change); 3 (copy number) NRAS 1.364 (fold change); 3 (copy number) ERBB2 1.537 (fold change); 3 (copy number) BRCA1 1.663 (fold change); 4 (copy number) RPS6KB1 1.723 (fold change); 4 (copy number)
Autopsy tissue	Lymph node metastasis	TP53 (ENST00000269305.4); exon 8; c.833C>T; p.P278L; VAF 77; 382/494; pathogen; cell cycle	0.8 mut/Mb (1.27 Mb)–TMB-low	3.45% (4/116)–MSS	NRAS 1.399 (fold change); 3 (copy number)
	Lung lesion	TP53 (ENST00000269305.4); exon 8; c.833C>T; p.P278L; VAF 73; 360/495; pathogen; cell cycle	1.6 mut/Mb (1.27 Mb)–TMB-low	4.3% (5/116)–MSS	
	Liver metastasis	TP53 (ENST00000269305.4); exon 8; c.833C>T; p.P278L; VAF 74; 257/345; pathogen; cell cycle	2.4 mut/Mb (1.27 Mb)–TMB-low	5.17% (3/58)–MSS	NRAS 1.557 (fold change); 4 (copy number) MDM4 1.478 (fold change); 4 (copy number) RICTOR 1.662 (fold change); 4 (copy number) FGF10 1.504 (fold change); 4 (copy number)
	Brain metastasis 1	TP53 (ENST00000269305.4); exon 8; c.833C>T; p.P278L; VAF 93; 603/949; pathogen; cell cycle	1.6 mut/Mb (1.27 Mb)–TMB-low	5.26% (5/116)–MSS	NRAS 1.520 (fold change); 4 (copy number) MDM4 1.645 (fold change); 4 (copy number) PIK3CB 1.356 (fold change); 4 (copy number) RICTOR 1.705 (fold change); 4 (copy number) FGF10 1.551 (fold change); 4 (copy number)
	Brain metastasis 2	TP53 (ENST00000269305.4); exon 8; c.833C>T; p.P278L; VAF 83; 721/871; pathogen; cell cycle	0.8 mut/Mb (1.27 Mb)–TMB-low	3.33% (4/116)–MSS	NRAS 1.441 (fold change); 3 (copy number) MDM4 1.458 (fold change); 3 (copy number)

TMB = tumor mutational burden; MSI = microsatellite instability; CNV = copy number variation; NGS = next-generation sequencing; VAF = variant allele frequency (in %); MSS = microsatellite-stable.

To secure high tumor cell content, lesions were, if necessary, macrodissected.

#### Longitudinal Profiling via Liquid Biopsies Enables Sensitive Monitoring of Disease Burden and Dynamic Clonal Evolution

In line with tumor tissue sequencing results (Table 1), the *TP53* c.833C>T of circulating tumor DNA represented the dominant tumor clone from initial diagnosis to end of treatment, thus serving as a surrogate marker for overall tumor burden over time (Fig. 1F). Clinical, radiologic, and metabolic response assessments aligned with the tumor burden dynamics, reflected by the circulating tumor DNA levels. Additionally, clonal complexity increased after 4 RPT cycles with the emergence of presumed subclonal aberrations in *APC, BRCA1, RET,* and *SLC18A3*.

## DISCUSSION

Our patient experienced an exceptional treatment response to fourth-line RPT with a prolonged progression-free survival of 6 mo and an overall survival of 8 mo (after previous failure of third-line therapy), compared with a progression-free survival of 1.6 mo and OS of 3.3 mo in a cohort of SCLC patients who received paclitaxel as third- or fourth-line therapy (18). This exceptional response indicates that SSTR-directed RPT is a feasible and effective promising therapeutic option.

Our immunohistochemical analyses are concordant with prior investigations showing that SSTR2 expression is closely linked to the SCLC-NEUROD1 subtype, suggesting a concomitant regulation (3). Some lesions showed a downregulation of immunohistochemical SSTR2 expression toward the end of treatment, which correlated with PET findings, in which the liver as well as adrenal gland metastases were not detectable at all. Interestingly, the SSTR2-negative lesions could not be assigned to another molecular SCLC subtype via immunohistochemistry, in line with a recent study with no evidence for transcriptional subtype conversion on therapy (19). However, other studies indicate intra- and intertumoral heterogeneity and molecular subtype switching (14, 20), partly linked to therapeutic pressure (21). As molecular subtyping could pave the way to more tailored treatment strategies in SCLC (22), further studies are necessary to address subtype heterogeneity, better understand subtype-dependent treatment (especially RPT response), and profile SSTR expression within molecularly defined SCLC subgroups.

As our liquid biopsy results indicate some degree of subclonal heterogeneity, it is reasonable that besides SSTR2 downregulation, clonal dynamics and heterogeneity could also be a relevant reason for radioresistance and aggressive behavior.

## CONCLUSION

This case provides immunohistochemistry and correlating dual-tracer imaging as proof of SSTR downregulation as an evasion mechanism under SSTR-directed RPT. This unique investigation provides a clinicomolecular in-depth characterization of a SCLC patient undergoing SSTR-directed RPT.

## DISCLOSURE

No potential conflict of interest relevant to this article was reported.

## Notes

KEY POINTS **QUESTION:** How does SSTR-directed peptide receptor RPT influence the tumor biology of SSTR-expressing SCLC? **PERTINENT FINDINGS:** Here, we report the case of a patient with therapy-refractory and advanced SCLC, who showed an exceptional response to SSTR-directed RPT. By performing a comprehensive translational work-up consisting of histopathologic, immunohistochemical, molecular pathology, and imaging analyses at different time points during treatment and after death, and especially of lesions with discordant tracer uptake, we could show that SSTR downregulation of some lesions seems to be an evasion mechanism under SSTR-guided RPT. Besides SSTR2 downregulation, clonal heterogeneity could be also a potential reason for therapy and radioresistance. **IMPLICATIONS FOR PATIENT CARE:** This unique investigation for a clinical–molecular understanding of novel treatment paradigms in SCLC may provide the basis for future individualized treatment designs for this devastating disease.

## References

[1] Gazdar AF Bunn PA Minna JD. Small-cell lung cancer: what we know, what we need to know and the path forward. Nat Rev Cancer. 2017;17:725–737. 29077690 10.1038/nrc.2017.87

[2] Dingemans AMC Früh M Ardizzoni A; ESMO Guidelines Committee. Small-cell lung cancer: ESMO Clinical Practice Guidelines for diagnosis, treatment and follow-up. Ann Oncol. 2021;32:839–853. 33864941 10.1016/j.annonc.2021.03.207 PMC9464246

[3] Chan JM Quintanal-Villalonga Á Gao VR. Signatures of plasticity, metastasis, and immunosuppression in an atlas of human small cell lung cancer. Cancer Cell. 2021;39:1479–1496.e18. 34653364 10.1016/j.ccell.2021.09.008 PMC8628860

[4] Tsuta K Wistuba II Moran CA. Differential expression of somatostatin receptors 1-5 in neuroendocrine carcinoma of the lung. Pathol Res Pract. 2012;208:470–474. 22770972 10.1016/j.prp.2012.05.014

[5] Strosberg J El-Haddad G Wolin E.; NETTER-1 Trial Investigators. Phase 3 trial of ^177^Lu-Dotatate for midgut neuroendocrine tumors. N Engl J Med. 2017;376:125–135. 28076709 10.1056/NEJMoa1607427 PMC5895095

[6] Strosberg JR Caplin ME Kunz PL.; NETTER-1 investigators. ^177^Lu-Dotatate plus long-acting octreotide versus high-dose long-acting octreotide in patients with midgut neuroendocrine tumours (NETTER-1): final overall survival and long-term safety results from an open-label, randomised, controlled, phase 3 trial. Lancet Oncol. 2021;22:1752–1763. 34793718 10.1016/S1470-2045(21)00572-6

[7] Singh S Halperin DM Myrehaug S. [^177^Lu]Lu-DOTA-TATE in newly diagnosed patients with advanced grade 2 and grade 3, well-differentiated gastroenteropancreatic neuroendocrine tumors: primary analysis of the phase 3 randomized NETTER-2 study [abstract]. J Clin Oncol. 2024;42(suppl):LBA588.

[8] Sommer S Schmutz M Hildebrand K. Concept and feasibility of the Augsburg longitudinal plasma study (ALPS): a prospective trial for comprehensive liquid biopsy-based longitudinal monitoring of solid cancer patients. J. Lab. Medicine. 2024;48:107–119.

[9] Zaknun JJ Bodei L Mueller-Brand J. The joint IAEA, EANM, and SNMMI practical guidance on peptide receptor radionuclide therapy (PRRNT) in neuroendocrine tumours. Eur J Nucl Med Mol Imaging. 2013;40:800–816. 23389427 10.1007/s00259-012-2330-6 PMC3622744

[10] Buechner P Hinderer M Unberath P. Requirements analysis and specification for a molecular tumor board platform based on CBIoPortal. Diagnostics. 2020;10:93. 32050609 10.3390/diagnostics10020093 PMC7167859

[11] Metzger P Hess ME Blaumeiser A. MIRACUM-Pipe: an adaptable pipeline for next-generation sequencing analysis, reporting, and visualization for clinical decision making. Cancers (Basel). 2023;15:3456. 37444566 10.3390/cancers15133456 PMC10340358

[12] Li MM Datto M Duncavage EJ. Standards and guidelines for the interpretation and reporting of sequence variants in cancer: a joint consensus recommendation of the Association for Molecular Pathology, American Society of Clinical Oncology, and College of American Pathologists. J Mol Diagn. 2017;19:4–23. 27993330 10.1016/j.jmoldx.2016.10.002 PMC5707196

[13] Baine MK Hsieh MS Lai WV. SCLC subtypes defined by ASCL1, NEUROD1, POU2F3, and YAP1: a comprehensive immunohistochemical and histopathologic characterization. J Thorac Oncol. 2020;15:1823–1835. 33011388 10.1016/j.jtho.2020.09.009 PMC8362797

[14] Megyesfalvi Z Barany N Lantos A. Expression patterns and prognostic relevance of subtype-specific transcription factors in surgically resected small-cell lung cancer: an international multicenter study. J Pathol. 2022;257:674–686. 35489038 10.1002/path.5922 PMC9541929

[15] Liebich A Enke JS Reitsam NG. SSTR antagonists as theranostic option in small cell lung cancer. Am J Respir Crit Care Med. October 4, 2023 [Epub ahead of print]. 10.1164/rccm.202303-0385IM 37792404

[16] Schiavo Lena M Partelli S Castelli P. Histopathological and immunophenotypic changes of pancreatic neuroendocrine tumors after neoadjuvant peptide receptor radionuclide therapy (PRRT). Endocr Pathol. 2020;31:119–131. 32361926 10.1007/s12022-020-09623-4

[17] Lapa C Hänscheid H Wild V. Somatostatin receptor expression in small cell lung cancer as a prognostic marker and a target for peptide receptor radionuclide therapy. Oncotarget. 2016;7:20033–20040. 26936994 10.18632/oncotarget.7706 PMC4991436

[18] Von Eiff D Bozorgmehr F Chung I. Paclitaxel for treatment of advanced small cell lung cancer (SCLC): a retrospective study of 185 patients. J Thorac Dis. 2020;12:782–793. 32274145 10.21037/jtd.2019.12.74 PMC7139030

[19] George J Maas L Abedpour N. Evolutionary trajectories of small cell lung cancer under therapy. Nature. 2024;627:880–889. 38480884 10.1038/s41586-024-07177-7 PMC10972747

[20] Ireland AS Micinski AM Kastner DW. MYC drives temporal evolution of small cell lung cancer subtypes by reprogramming neuroendocrine fate. Cancer Cell. 2020;38:60–78.e12. 32473656 10.1016/j.ccell.2020.05.001 PMC7393942

[21] Stewart CA Gay CM Xi Y. Single-cell analyses reveal increased intratumoral heterogeneity after the onset of therapy resistance in small-cell lung cancer. Nat Cancer. 2020;1:423–436. 33521652 10.1038/s43018-019-0020-z PMC7842382

[22] Schwendenwein A Megyesfalvi Z Barany N. Molecular profiles of small cell lung cancer subtypes: therapeutic implications. Mol Ther Oncolytics. 2021;20:470–483. 33718595 10.1016/j.omto.2021.02.004 PMC7917449

